# Cryotherapy for Eccrine Poroma: A Case Report

**DOI:** 10.7759/cureus.36563

**Published:** 2023-03-23

**Authors:** Shahad Alkidaiwi, Fawaz H Aljehani, Sara Alharbi, Ethar Alsaedi

**Affiliations:** 1 Dermatology, King Abdulaziz Hospital, Makkah, SAU; 2 Medicine and Surgery, Umm Al-Qura University, Makkah, SAU

**Keywords:** treatment, cryotherapy, cryosurgery, eccrine poroma, adnexal tumors

## Abstract

Eccrine poroma (EP) is a benign adnexal tumor that is derived from acrosyringium, the intraepidermal eccrine duct of sweat glands. The standard treatment for eccrine poroma is complete excision. However, this case report highlights cryotherapy as one of the modalities in treating eccrine poroma. We present a case of a 33-year-old male patient who was a known case of generalized vitiligo since he was nine years old. During our skin checkup before starting him on phototherapy, we found a mass over the palmar aspect of the middle finger of the right hand that started to appear five years ago. The mass gradually increased in size, was painless, has no discharge, and was not associated with a history of trauma or infection. The review of systems was unremarkable. Skin examination revealed an asymptomatic, 2.0 × 1.5 cm-sized, solitary, collarette-encircled, dome-shaped, flesh-colored, non-pigmented, deep-red nodule protrusion from the palmar aspect of the middle finger of the right hand. Poroma was considered as the diagnosis, and a punch skin biopsy was performed to confirm the diagnosis and to roll out pyogenic granuloma, amelanotic melanoma, and porocarcinoma as differential diagnoses. A 3 mm punch skin biopsy was performed under local anesthesia and was found to be histologically consistent with eccrine poroma. Hence, cryosurgery was chosen based on histological favorable features. We used cryospray in a single session of 15 seconds in three applications, with five-second intervals in between (skin frosting recovery). Furthermore, the lesion was completely curative with a single session of cryotherapy. The patient followed up for one year without evidence of recurrence.

## Introduction

Eccrine poroma (EP) is a benign adnexal tumor that is derived from the acrosyringium, the intraepidermal eccrine duct of sweat glands [1]. Sweat gland tumors represent 1% of all primary skin tumors and approximately 10% of benign sweat gland tumors are poromas [2]. A poroma presents as a solitary, slow-growing, dome-shaped, vascular-appearing papule or nodule, most commonly exhibiting on the acral surfaces of the palms or soles [3-5]. Clinically, the condition resembles both benign and malignant diseases, including basal cell carcinoma, squamous cell carcinoma, and pyogenic granuloma [6]. Treatment is optional, as EP is considered a benign adnexal neoplasm. However, excision is the main treatment [6]. In this article, we report a case of EP that was treated with a single session of cryotherapy and was curative.

## Case presentation

**Figure 1:**
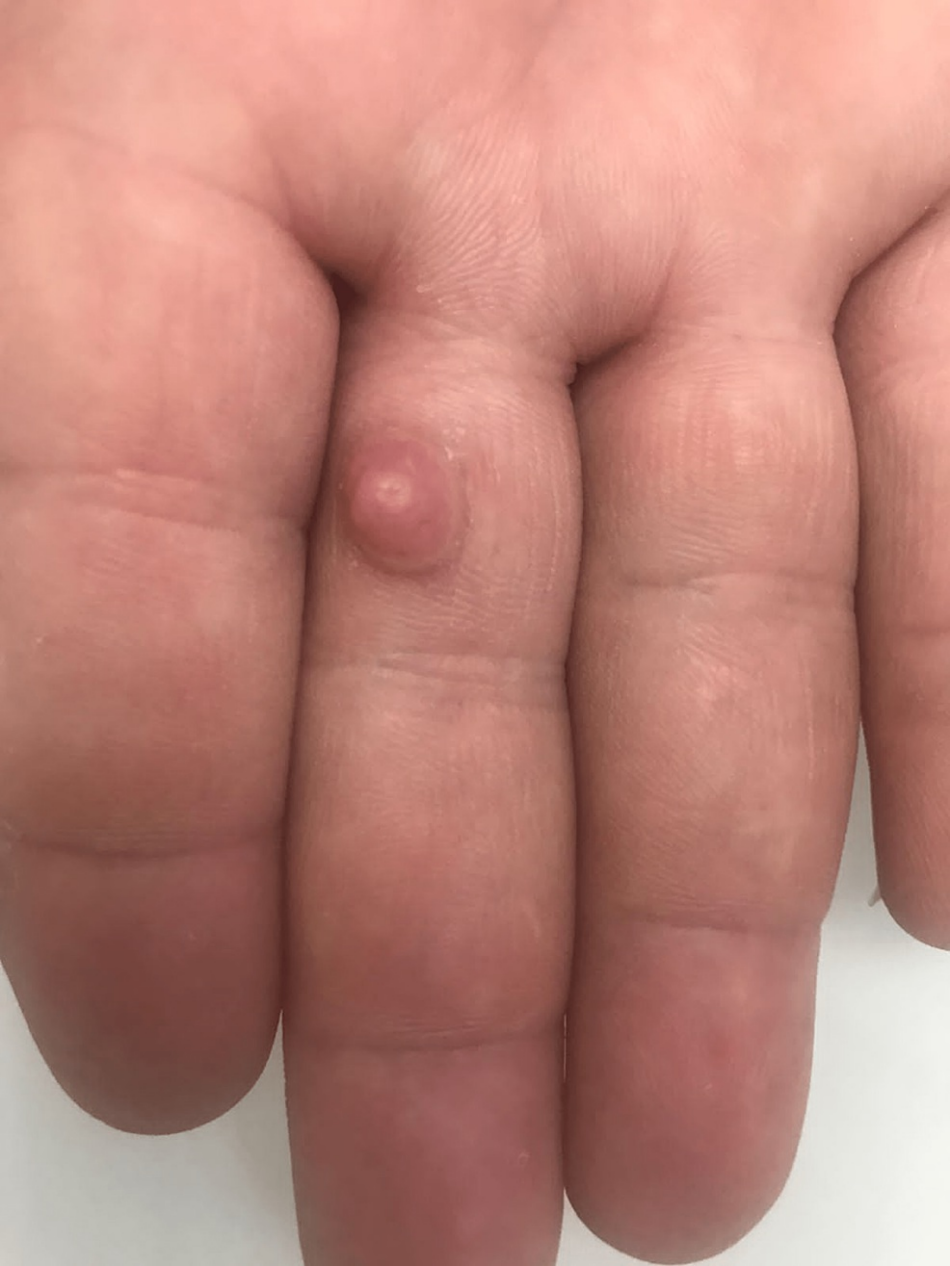
The image shows an erythematous nodule on the palmar aspect of the middle finger

A 33-year-old male patient, a known case of generalized vitiligo since he was nine years old, was recently started on narrowband ultraviolet B (NB UVB) and then shifted to excimer laser because the patient could not tolerate excessive heat. During the skin checkup before the beginning of his phototherapy, we found a mass over the palmar aspect of the middle finger of the right hand that started to appear five years ago. The mass gradually increased in size, was painless, has no discharge, and was not associated with a history of trauma or infection. The review of systems was unremarkable. Examination revealed an asymptomatic, 2.0 × 1.5 cm-sized, solitary, collarette-encircled, dome-shaped, flesh-colored, non-pigmented, deep-red nodule protrusion from the palmar aspect of the middle finger of the right hand (Figure 1).

**Figure 2:**
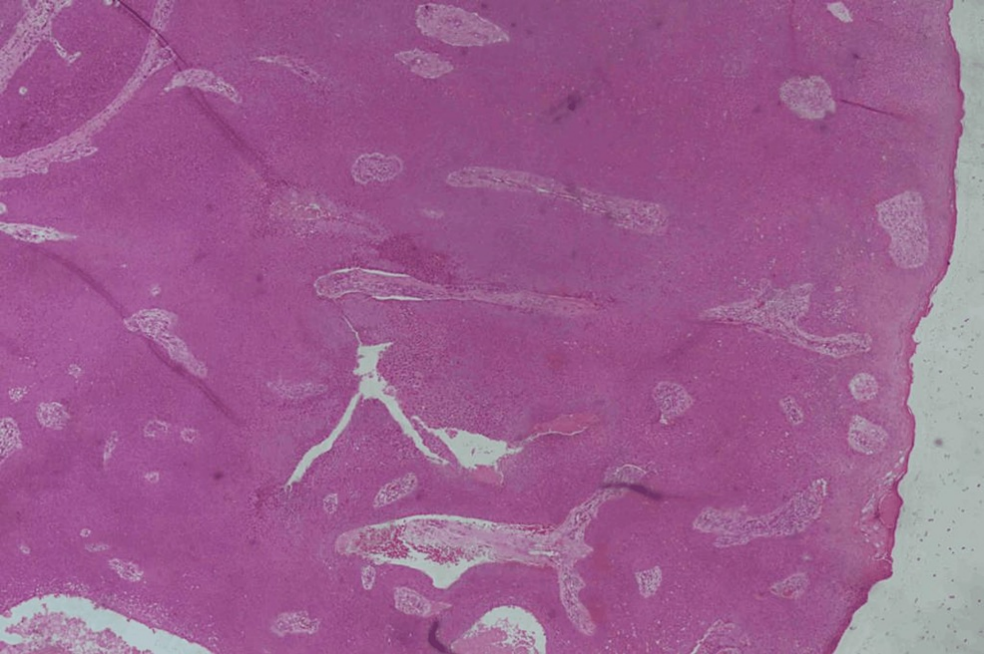
Pathology A 3 mm punch skin biopsy revealed a well-circumscribed, dermal proliferation of small, uniform, cuboidal cells with basophilic round nuclei and compact eosinophilic cytoplasm. Broad anastomosing bands of epithelial cells extending deep into the dermis (hematoxylin & eosin stain; original magnification, x20).

Poroma was considered the diagnosis, and a punch skin biopsy was performed to confirm the diagnosis and to roll out pyogenic granuloma, amelanotic melanoma, and porocarcinoma as differential diagnoses. A 3 mm punch skin biopsy was performed under local anesthesia and was found to be histologically consistent with eccrine poroma (Figure 2).

**Figure 3:**
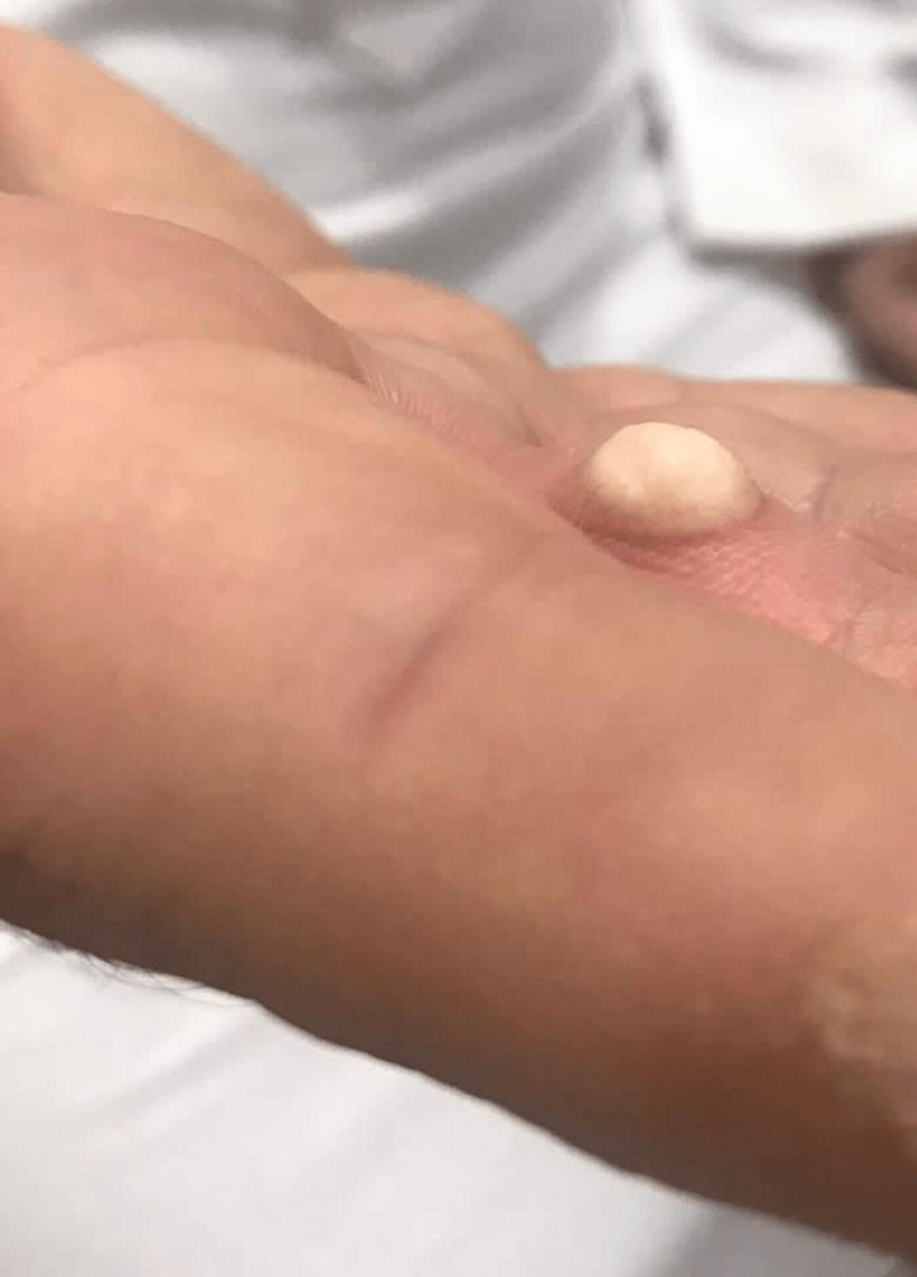
The image shows the lesion immediately after the cryotherapy

Hence, cryosurgery was chosen based on the histologically favorable features. We used cryospray in a single session of 15 seconds in three applications with five-second intervals in between (skin frosting recovery) (Figure 3).

**Figure 4:**
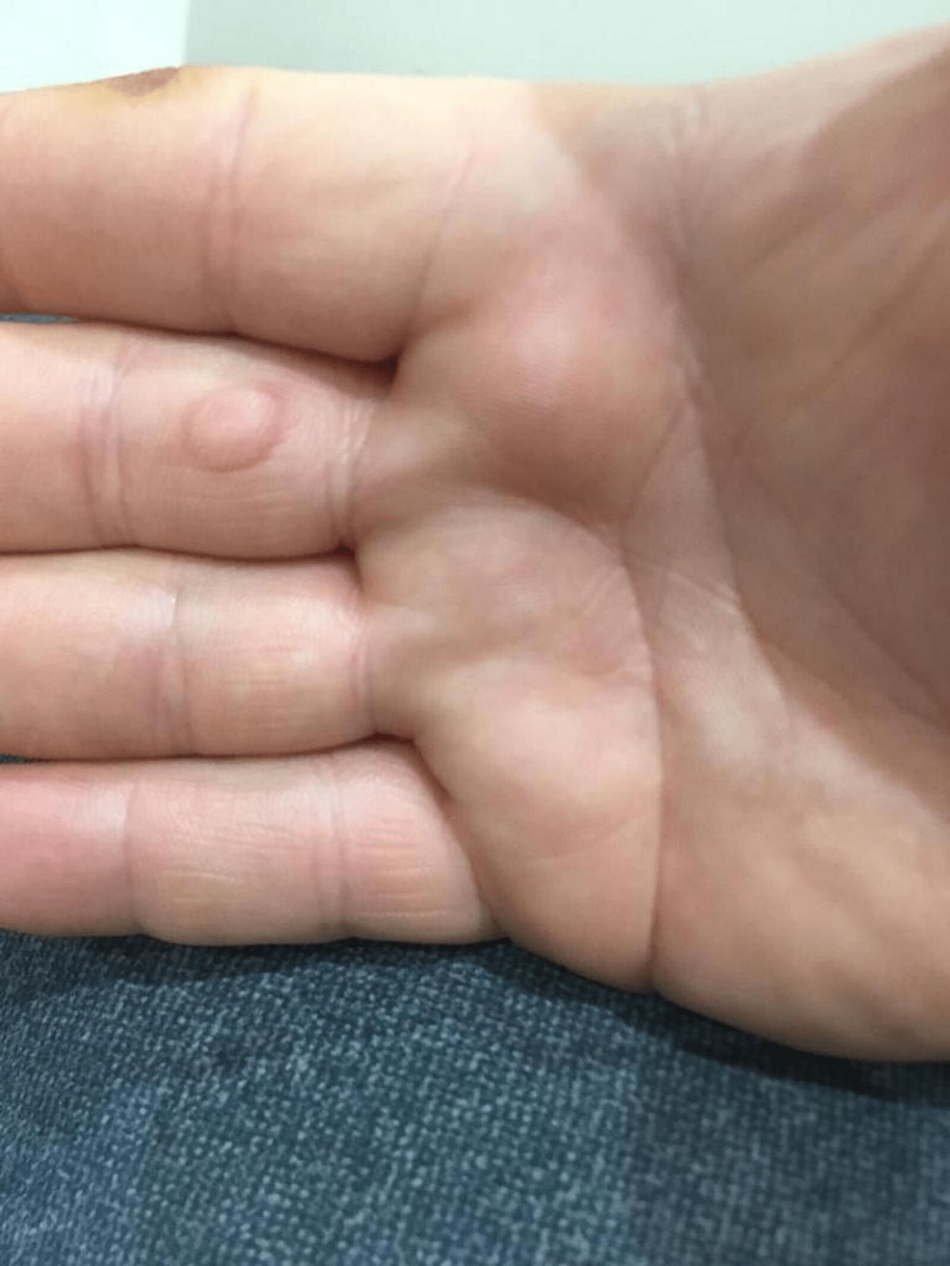
The image shows the lesion after a one-year follow-up

Furthermore, the lesion was completely curative with a single session of cryotherapy. Then, the patient followed up for one year without evidence of recurrence (Figure 4).

## Discussion

Our case study is unique in the treatment modality since the lesion was resolved after a single session of cryotherapy and followed up for one year without evidence of recurrence. The standard for EP is complete excision to prevent recurrence [7]. On the other hand, the most common outcome of cryosurgery is local changes in skin pigmentation, erythema, and edema [8]. Moreover, scarring is an uncommon but potential side effect of cryotherapy [8]. Permanent hair loss is also one of the expected adverse events of cryosurgery when performed on the scalp or in other hairy areas [8]. Consequently, the depth of freezing has a significant impact on healing time; hence, lesions that have been frozen more deeply will take longer to heal [8]. A previous case report of poromatosis in pregnancy was published in February 2012 [9]. Comparatively, their patient had eight eruptive painful lesions that were smaller and developed in the third trimester of pregnancy [9]. The EP diagnosis was made through a histopathological examination of excisional punch biopsies from two lesions [9]. The remaining six lesions were successfully treated with cryotherapy, with no evidence of recurrence at six months postpartum [9]. However, the number of cryotherapy sessions was not mentioned [9]. Additionally, another case report was published in July-August 2015 about eccrine poroma with the typical clinical and histological findings of EP [10]. There was a difference, however, in our cases with regard to the treatment modality [10]. In the literature, nearly all the cases were treated with surgical excision to prevent the risk of recurrence or progression to malignancy [10]. This is in comparison to our patient who was treated with a single session of cryotherapy and followed up for one year without evidence of recurrence.

## Conclusions

For the treatment of benign and solitary eccrine poroma, cryotherapy is an efficient and well-tolerated method whose advantages include quick execution, lack of need for local anesthesia, and low cost. However, there are no established guidelines for when cryotherapy should be applied to various parts of the body and for how long to freeze and thaw. In this context, randomized controlled clinical trials (RCTs) are required to improve the procedure and reduce the risk of over- or under-treatment.
